# Physiological Characteristics of Photosynthesis in Yellow-Green, Green and Dark-Green Chinese Kale (*Brassica oleracea* L. var. *alboglabra* Musil.) under Varying Light Intensities

**DOI:** 10.3390/plants9080960

**Published:** 2020-07-30

**Authors:** Kuan-Hung Lin, Feng-Chi Shih, Meng-Yuan Huang, Jen-Hsien Weng

**Affiliations:** 1 Department of Horticulture and Biotechnology, Chinese Culture University, Taipei 111, Taiwan; rlin@faculty.pccu.edu.tw; 2 Department of Life Sciences, National Chung-Hsing University, Taichung40227, Taiwan; a86229265@yahoo.com.tw

**Keywords:** chlorophyll fluorescence, energy dissipation, light response, photoinhibition, photosynthesis efficiency, photosynthetic photon flux density, pigment

## Abstract

The objective of this work was to study physiological characteristics and photosynthetic apparatus in differentially pigmented leaves of three Chinese kale cultivars. Chlorophyll (Chl) fluorescence and photochemical reflectance index (PRI) measurements in green, yellow-green, and dark-green cultivars in response to varying light intensities. As light intensity increased from 200 to 2000 photosynthetic photon flux density (PPFD), fraction of light absorbed in photosystem (PS) II and PRI values in all plants were strongly lowered, but fraction of light absorbed in PSII dissipated via thermal energy dissipation and non-photochemical quenching (NPQ) values in all plants wereremarkably elevated.When plants were exposed to 200 PPFD, the values of fraction of light absorbed in PSII, utilized in photosynthetic electron transport(*p*), andfraction of light absorbed excitation energy in PSII dissipated via thermal energy dissipation (*D*), remained stable regardless of the changes in levels of Chla + b. Under 800 and 1200 PPFD, the values of *p* and electron transport rate (ETR) decreased, but *D* and NPQ increased as Chla + bcontent decreased, suggesting that decrease inChla + bcontent led to lower PSII efficiency and it became necessary to increase dissipate excess energy. On the contrary, in 2000 PPFD, leaves with lower Chla + bcontent had relatively higher *p* and electron transport rate (ETR) values and lower *D* level, as well as tended to increase more in NPQ but decrease more in PRI values. The consistent relations between PRI and NPQ suggest that NPQ is mainly consisted ofthe xanthophyll cycle-dependentenergy quenching.Yellow-green cultivar showed lower Chla + bcontent but high carotenoids/Chla + b ratio and had high light protection ability under high PPFD. The precise management of photosynthetic parameters in response to light intensity can maximize the growth and development of Chinese kale plants.

## 1. Introduction

Photosynthesis is biochemically regulated to maintain the balance between the rates of its component processes and concentrations of metabolites in response to environmental changes. Eco-physiological studies require knowledge of the photosynthetic rates of plants under different environmental conditions and a broad range in light intensity. Plants respond to sudden and sustained fluctuations in light intensities, partly throughtheir molecular redox-signaling transduction mechanisms in the chloroplast [1]. Light intensity not only affects plant growth and biochemical characteristics, but also associates with the photosynthetic efficiency of plants. The study of photosynthesis irradiance relationships is a basic aspect of plant physiological research and is important for managing various species; photosynthetic light responses can be used to assess the ability to capture light and understand the optimal habitat light intensity conditions of plants [2].

Both chlorophyll fluorescence (ChlF) and reflectance spectroscopy (i.e., photochemical reflectance index, PRI) are noninvasive techniques andoften used in physiologicalstudies to investigate a plant′s response to various abiotic and biotic stresses in controlled environments and in the field [3]. The measurements of ChlF and PRI are simple and reliable methods for estimating the photosynthetic rate [4,5]. However, previous research also found that ChlF parameters were affected by the amount of photosynthetic pigments [6]. The effect of pigments on ChlF and PRI in Chinese kale species with different leaf colors subjected todifferent light intensities has not yet been examined.

Chinese kale (*Brassica oleracea* L. var. *alboglabra*) is a crucifer vegetable crop grown in Southeast Asian countries [7]. There are many cultivars with varied leaf colors, includingyellow-green, green, and dark-green. Most of the plant materials with low Chl contentscan be collected from nitrogen deficiency or restricted treatments, wheresome materials, such as Chl-deficientspecies or mutants, can be used for photosynthesis measurements under normal nutrition or full fertilization conditions.Therefore, it is worth to study the effect of pigments on ChlF and PRI in varied leaf colors of Chinese kale species under full and restrictedfertilization conditions at variousphotosynthetic photon flux densities (PPFD). However, there is limited information available regarding ChlF and spectral reflectanceof these plants under various light intensities. Understanding the photosynthetic characteristics of the Chinese kale would benefit field cultivation management and inform the relationship between leaf color and light energy distribution and utilization. The hypothesis of this study was that ChlFparameters might exhibit distinguishable differences among yellow-green, green, and dark-green Chinese kale plants under different light intensities since the pigment composition of their leaves differs. ChlF and PRI indices would be changed in varied leaf colors with different Chl contents in response to different PPFDs.The aim of this study was to determine the actual state of the photosynthetic apparatus in differently pigmented leaves of three Chinese kale cultivars. The appearance of the capture, transfer, and dissipation of excitation energy were detected by ChlF measurements and PRI values in green, yellow-green, and dark-green cultivars in response to varying light intensities. ChlF and PRI can be considered selection indexes for examining the growth of Chinese kaleplants at specific and optimal light intensities under artificial light illuminations. The relationship of ChlF and PRI indices with Chla + band carotenoids (Car) can be used for eco-physiological research in Chinese kaleplants. The precise management of ChlF and PRI parameters in response to light irradiances may hold promise for maximizing the economic efficiency of the growth, development, and pigment potential of Chinese kaleplants grown in controlled environments.

## 2. Results

### 2.1. Influence of Light Intensity on Chl a + band the Fluorescence Parameters of Chinese Kale Plants

**Figure 1:**
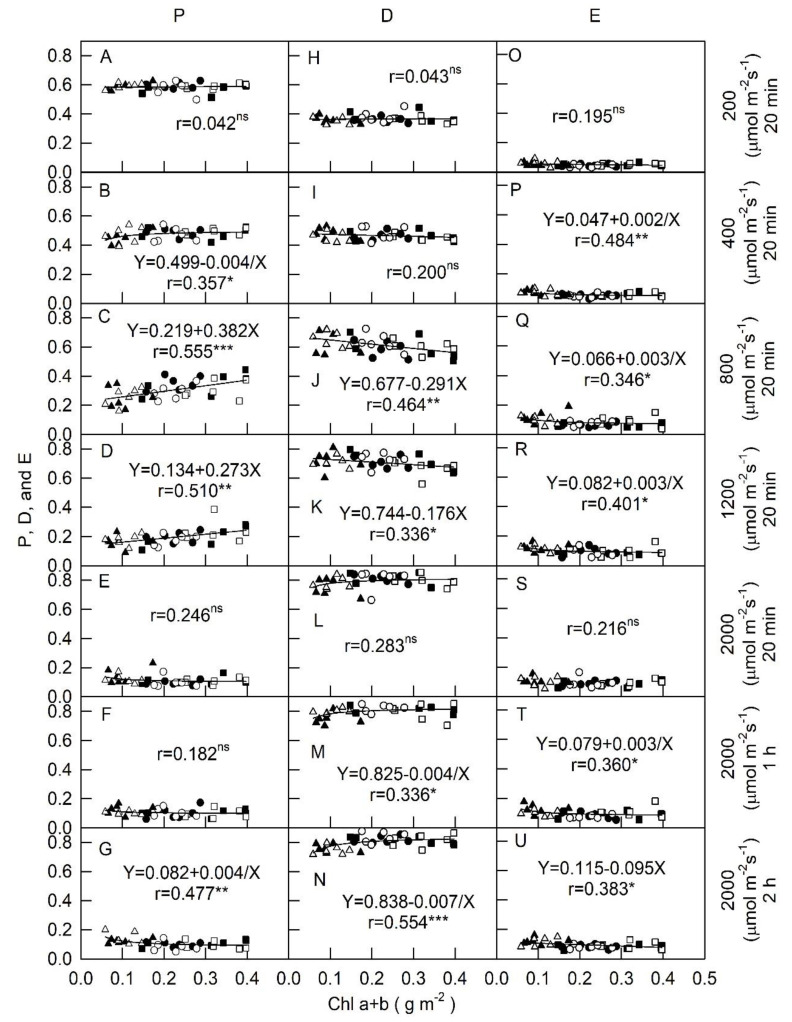
Relationships among Chla + b content, fraction of light energy absorbed in photosystem II that is utilized in photochemistry (*p*) (panels A–G), dissipated thermally (*D*) (panels H–N), and excess energy (*E*) (panel O–U) in yellow-green (triangle, ▲ and △), green (circle, ● and ○), and dark-green (square, ■ and □) foliage cultivars of Chinese kale with respect to different light intensity treatments (PPFD of 200, 400, 800, and 1200 μmol m^−2^s^−1^ for 20 min, and 2000 μmol m^−2^ s^−1^ conditions for 20 min, 1 h, and 2 h). Black and white represents a plant treated with full fertilization (100 mL of 0.1%NH_4_NO_3_ and K_2_HPO_4_ applied twice weekly) and restricted fertilization (50 mL of 0.1% NH_4_NO_3_ and K_2_HPO_4_ applied once weekly), respectively. Each symbol represents the average of one leaf on one plant and 36 plants were randomly selected from each treatment. Each Chl index was calculated using yellow-green (triangle, ▲ and △), green (circle, ● and ○), and dark-green (square, ■ and □) with leaf data (*n* = 36) from the model′s validation datasets. The correlation coefficient (r) and significance of the regression are shown (* *p* < 0.05; ** *p* < 0.01; *** *p* < 0.001; ns, not significant).

**Figure 2:**
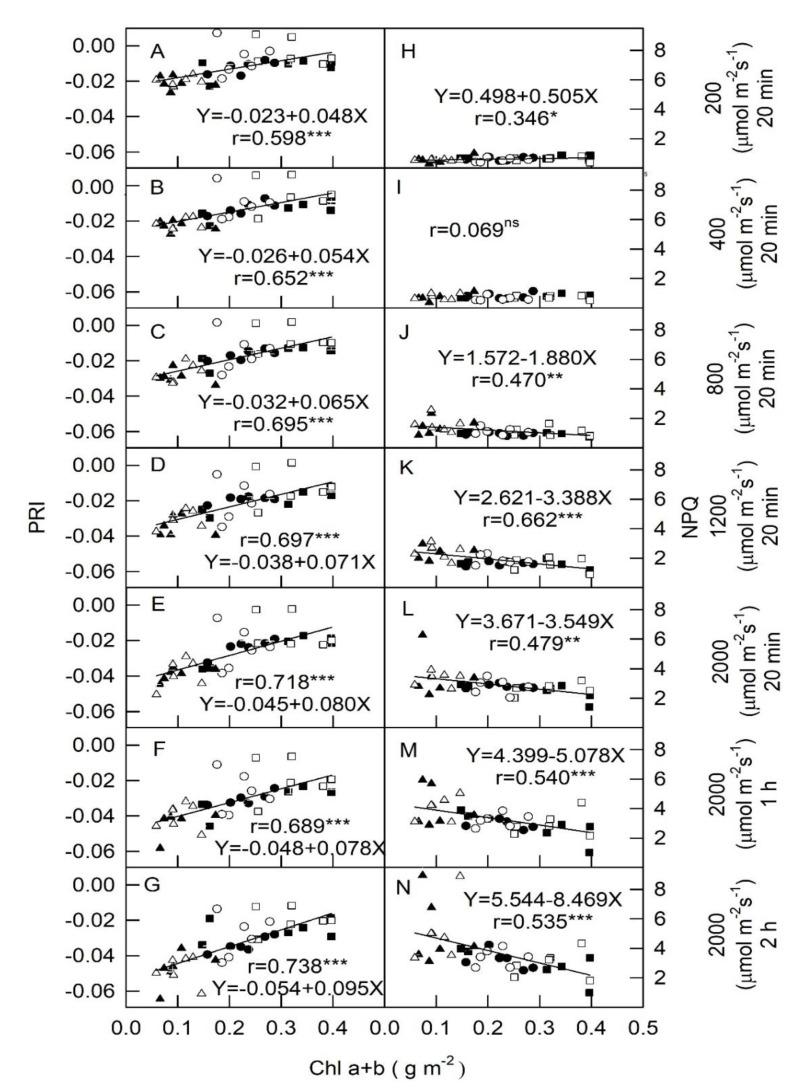
Relationships among Chla + b content, photochemical reflectance index (PRI) (panels **A**–**G**), and non-photochemical quenching (NPQ) (panels **H**–**N**) in yellow-green (triangle, ▲ and △), green (circle, ● and ○),and dark-green (square, ■ and □) foliage cultivars of Chinese kale with respect to different light intensity treatments (PPFD of 200, 400, 800, and 1200 μmol m^−2^ s^−1^ for 20 min, and 2000 μmol m^−2^ s^−1^conditions for 20 min, 1 h, and 2 h). Black and white represents a plant treated with full fertilization (100 mL of 0.1% NH_4_NO_3_ and K_2_HPO_4_ applied twice weekly) and restricted fertilization (50 mL of 0.1% NH_4_NO_3_ and K_2_HPO_4_ applied once weekly), respectively. Each symbol represents the average of one leaf on one plant and 36 plants were randomly selected from each treatment. Each Chl index was calculated using yellow-green (triangle, ▲ and △), green (circle, ● and ○), and dark-green (square, ■ and □) with leaf data (*n* = 36) from the model′s validation datasets. The correlation coefficient (r) and significance of the regression are shown (* *p* < 0.05; ** *p* < 0.01; *** *p* < 0.001; ns, not significant).

Figure 1 and Figure 2 show thatphotosynthetic electron transport (*p*) and PRI levels of all plantsdecreased, but thermal energy dissipation (*D*) and non-photochemical quenching (NPQ) values increased as light intensity increased from 200 to 2000 μmol m^−2^s^−1^ PPFD. In addition, relationships among *p*, *D*, excess energy (*E*), and PRI vs. Chla + b in all plants were varied with PPFDs. The *p*, *D*, and *E* values in all plants exposed to 200 μmol m^−2^s^−1^were approximately 0.6, 0.4, and 0.1, respectively, and displayed no variation with the change of Chla + b (Figure 1A,H,O). There were positive and significant correlations at r = 0.357, 0.555, and 0.510 between *p* and Chla + b in all tested plants in response to 400, 800, and 1200 μmol m^−2^s^−1^, respectively (Figure 1B–D). The value of *p* in all plants under 2000 μmol m^−2^s^−1^ for all irradiation times was below 0.2 (Figure 1E–G) and displayed no relationship between *p* and Chl a + b except for a negative correlation (r = 0.477, *p* < 0.05) between *p* and Chl a + b under 2000 μmol m^−2^s^−1^ for 2 h (Figure 1G). No correlations were observed between *D* and Chl a + b in all plants under 200, 400, and 2000 μmol m^−2^s^−1^ for 20 min (Figure 1I,L). Nevertheless, *D* was significantly and negatively correlated with Chla + b at PPFDs of 800 and 1200 μmol m^−2^ s^−1^ (r = 0.464 and 0.336, respectively; Figure 1J,K). The values of *D* werenon-linearly significant and positively correlated with Chla + b at r = 0.336 and 0.554 under 2000 μmol m^−2^s^−1^ for 1 h and 2 h, respectively (Figure 1M,N). All E values were weakly but significantly and negatively correlated to Chl a + b (r = 0.484–0.346, Figure 1P–R,T,U) in response to all PPFDs, except for no relationships being observed under 200 and 2000 μmol m^−2^ s^−1^ for 20 min (Figure 1O,S), indicating that a lower Chl a + b content resulted in an increased *E* and produced excess energy in PSII in response to different PPFDs. The trend of *D* against Chla + b in all plants was opposite to *p* values in response to medium PPFDs (800 and 1200 μmol m^−2^s^−1^) and high PPFD (2000 μmol m^−2^s^−1^), where medium and high PPFDs yielded relatively higher *D* values from 0.6 to 0.8 readings (Figure 1J–N) compared to *p* values (0.1–0.4, Figure 1C–G). These results demonstrate that leaves with lower Chl content showed lower *p* and led to increasing *D* to dissipated more excess light energy in PSII under medium PPFD condition. On the contrary, in high PPFD condition, leaves with lower Chla + bcontent had relatively higher *p* value and lower *D* level. The *E* values were less affected by Chl a + b under all tested PPFDs due to relatively low values.

**Figure 3:**
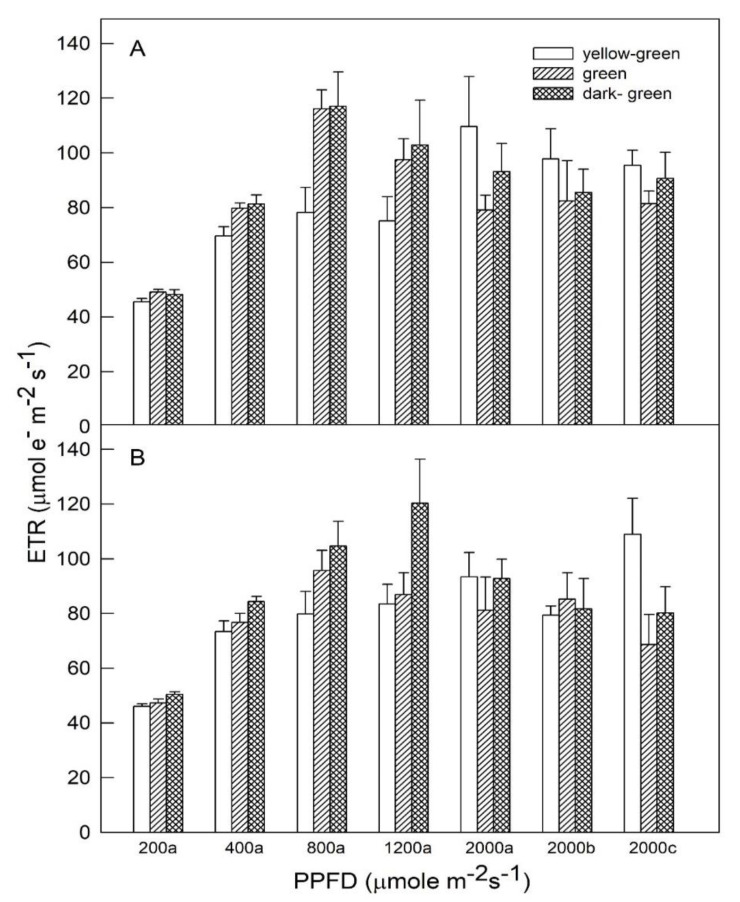
The responses of electron transportrate (ETR) to photosynthetic photon flux density (PPFD) in yellow-green (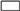), green (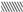), and dark-green (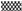) foliage cultivars of Chinese kale cultivated under (**A**) full fertilization treatment (100 mL of 0.1% NH_4_NO_3_ and K_2_HPO_4_ applied twice weekly) and (**B**) restricted fertilization treatment(50 mL of 0.1% NH_4_NO_3_ and K_2_HPO_4_ applied once weekly). Measurements were made in different light intensities at 200, 400, 800, and 1200 μmol m^−2^ s^−1^ for 20 min, and 2000 μmol m^−2^ s^−1^ PPFD conditions for 20 min (a), 1 h (b), and 2 h (c). Vertical bars indicate standard deviations (*n* = 6).

Correlations between Chl a + b and PRI measurements of all plant leaves with all fertilizer treatments in response to PPFD conditions exhibited significant and positive r values (0.598–0.738, *p* < 0.001, Figure 2A–G).In general, Chl a + b and PRI values in dark-green Chinese kale were relatively higher than in green and yellow cultivars in response to all PPFDs. As PPFD increased from 200 to 2000 μmol m^−2^s^−1^, PRI values decreased from −0.02 to −0.05 in response to lower leaf Chl a + b content (<0.1 gm^−2^), butincreased from −0.005 to −0.02 in higher leaf Chl a + b content (≒0.4 gm^−2^). Thus, the slope of the linear equation in PRI vs. Chl a + b increased from 0.048 to 0.095 as light intensity increased from 200 to 2000 μmol m^−2^s^−1^ PPFD. NPQ vs. Chl a + b variations in response to light intensities inall plants are demonstrated in Figure 2H–N. Data from all tested cultivars with all fertilization treatments show that the relationship between NPQ and Chl a + b was significantly and negatively correlated (r = 0.470–0.662) in all plants under medium and high PPFD conditions (Figure 2K–N). Nevertheless, weakly positive (r = 0.346, *p* < 0.05) and no insignificant correlations were detected between NPQ and Chla + b at 200 and 400 μmol m^−2^s^−1^, respectively (Figure 3H,I). The trend of NPQ vs. Chl was opposite to PRI vs. Chl. As PPFD increased from 200 to 2000 μmol m^−2^s^−1^,NPQ values increased from 1 to 5 in response to lower leaf Chl a + b content (<0.1 gm^−2^), but NPQ values only increased from 1 to 2 in higher leaf Chl a + b content (≒0.4 gm^−2^), suggesting that leaves of lower Chl content resulted in increasing NPQ and dissipating more excess light energy under medium (800 and 1200 μmol m^−2^s^−1^) and high (2000 μmol m^−2^s^−1^) PPFD conditions. No effects of fertilization (FF and RF) on Chl a + b vs. ChlF and PRI in all plants at all PPFDs were detected in this study.

### 2.2. ETR Values of Three Chinese Kale Cultivars underVariousLight Intensity Conditions

ETR values were analyzed under various light intensities (Figure 3). For full fertilization (panel A), the ETR of all plants under 200 μmol m^−2^s^−1^ PPFD did not show any differences. The highest ETR values at 118 μmol em^−2^s^−1^ were found in green and dark-green plants under 800 μmol m^−2^s^−1^ PPFD, and both green and dark-green leaves had significantly higher ETR values comparedto yellow leaves under 400, 800, and 1200 μmol m^−2^s^−1^ PPFDs. However, relatively higher ETR values were found in yellow-green plants subjected to 2000 μmol m^−2^s^−1^ PPFDs compared to green and dark-green plants. Furthermore, ETR values of all plants grown under restricted fertilization (panel B) were near to those of full fertilization condition. Dark-green plants had the lowest ETR value (≒50 μmol em^−2^s^−1^) at 200 μmol m^−2^s^−1^ PPFD and the value increased greatly to the peak (120 μmol em^−2^s^−1^) at 1200 μmol m^−2^s^−1^ PPFD, then the value decreased slowly to 80 μmol em^−2^s^−1^ at 2000 μmol m^−2^s^−1^ PPFD for 2 h. Both green and yellow-green plants showed no significant differences in ETR under all light irradiances, except that yellow-green leaves had significantly higher ETR value (110 μmol em^−2^s^−1^) than other plants at 2000 μmol m^−2^s^−1^ PPFD for 2 h.

### 2.3. Relationships betweenChl a + b and Car/Chl a + b

**Figure 4:**
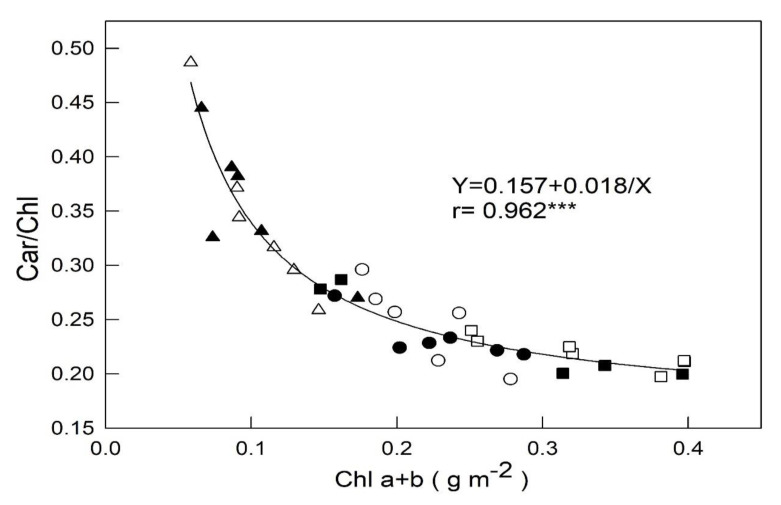
Relationships between Chla + b content and Car/Chla + bin yellow-green (triangle, ▲ and △), green (circle, ● and ○), and dark-green (square, ■ and □) foliage cultivars of Chinese kale. Black and white represents a plant beingtreated with full fertilization (100 mL of 0.1% NH_4_NO_3_ and K_2_HPO_4_ applied twice weekly) and restricted fertilization (50 mL of 0.1% NH_4_NO_3_ and K_2_HPO_4_ applied once weekly), respectively. Each symbol represents the average of one leaf on one plant and 36 plants were randomly selected from each treatment. Each Chl index was calculated using yellow-green (triangle, ▲ and △), green (circle, ● and ○), and dark-green (square, ■ and □) with leaf data (*n* = 6) from the model′s validation datasets. The correlation coefficient (r) and significance of the regression are shown (*** *p* < 0.001).

Relationship betweenChla + band Car/Chla + bin varied leaves is presented in Figure 4. Regression analysis showed that Car/Chl was significantly, strongly, and positivelycorrelated with Chla + b at r = 0.962. Moreover, yellow-green leaf had low Chl a + b content and high Car/Chla + bratio.

## 3. Discussion

Routes for light energy absorbed by Chl molecules in photosynthetic tissue are used to drive photosynthetic processes, dissipate heat, and re-emit light energy such as ChlF [8]. Measuring the yield of ChlF gives specific information about photochemical efficiency and heat dissipation. Changes in ChlF can be used to quickly assess plant physiological responses during stress [9]. Yang et al. [10] studied seasonal relationships between ChlFand photosynthesis at ecosystem scales and showed how leaf ChlF was linked with canopy-scale solar-induced ChlF in a temperate deciduous forest, indicating that ChlF can be used to track photosynthetic rates at leaf, canopy, and ecosystem scales. Since PSII is thought to play a key role in the response of PS to environmental stress, the analysis of ChlF under varying light intensities might reflect PSII behavior. In this study, three Chinese kale cultivars with varied leaf colors were used to elucidate the characteristics of Chla + bas related to ChlF under PPFDs.

As light intensity increased from 200 to 2000 μmol m^−2^s^−1^ PPFD, lower *p* levels, as well as higher *D* and NPQ values were observed in all plants. Plants can absorb more photons under high irradiance intensities than can be used in carbon fixation reactions [11]. This excess absorbed light energy becomes a stressor and enhances the formation of reactive oxygen species (ROS) that can damage many cellular components and therefore cause a depression in photosynthetic efficiency, especially in PSII [12]. In our study, the excess energy in PSII increased, leading to increases in *D* and NPQ values and decreases in *p* values due to greater energy dissipation when plants were exposed to higher PPFDs. The relationship between Chla + b and ChlF in all plants were varied with PPFDs.When plants were subjected to low light intensities (200 μmol m^−2^s^−1^), the values of *p* and *D* remained stable regardless of the changes in levels of Chl a + b, indicating that allocation of absorbed light energy in PSII was less affected by Chla + b content under low PPFD. However, when these plants were measured under moderate light intensities (800 and 1200 μmol m^−2^s^−1^ PPFD), *p* values increased but *D* and *E* values decreased as Chl a + b content increased, suggesting that thermal energy dissipation took place more in antennae and that when low Chl content led to lower PSII efficiency it became necessary to increase D in order to dissipate excess energy. Any environmental and physiological factors resulting in decreased photosynthesis rates at a constant PPFD should be expected to have the potential to lead to a greater excess of absorbed light. It was reported that the leaves with lower Chl content showed a lower photosynthetic rate [6]. Thus, under moderate light intensities, leaves with low Chl content had low *p* values due to high energy dissipation.

ETR is the product of PSII efficiency and absorbed light anddescribes the relative rate of electron transport through PSII [13]. In C_4_ plants, photorespiration is restricted, absorbedphotons may be mostly used to drive the CO_2_ fixation, and PSII efficiency is always parallel to variation in quantum yield of CO_2_ fixation inmany cases [14,15]. In contrast, a significant correlation was found between ETR and gross photosynthetic rate under conditionsin C_3_ plants, which lessen the interference of photorespiration and other alternative pathways for electrons, such asnear-constant temperature, CO_2_ and O_2_ concentrations [16,17]. In our study, when plants were measuredat 400–1200 μmol m^−2^s^−1^ PPFD, plants with deeper leaf colortended to have higher ETR values for higher efficiency of photosynthesis (Figure 3). Nevertheless, when plants were measuredat 2000 μmol m^−2^s^−1^ PPFD, yellow-green leaves had higher *p* and ETR values. These results indicate that dark-green cultivarfavored moderate PPFDs while yellow-green cultivar was adapted under high light intensities. ETR was calculated from *p*, but why could the consistent relations between *p* and ETR not be found under different PPFDs? A possible reason is that ETR value was calculated from both *p* value and absorbed light, but plant leaves with higherChla + bcontent had higher light absorbance rates (i.e., the leaves had a light absorbance rate between 0.76 and 0.88 in the study).

Chinese kale contains phenolic components, antioxidants, and free radical scavenging properties, and has been the subject of chemical and biological studies [18,19].The final goal of the study was to develop a year-round production system for healthy, fresh, nutritional, and economically feasible Chinese kale plants produced in a controlled environment. An optimal strategy of light intensity regulation will help in designing growth chambers and greenhouse light environments to obtain maximum economic benefits for growing Chinese kale plants and also improving field cultural practices for growing these plants in hydroponics as pesticide-free vegetables. Therefore, these ChlFcomponents can be used as indices to characterize the physiology of Chinese kale plants in response to different PPFDs.

Light intensity not only influences the accumulation of photosynthetic pigments, but also mediates photo-physiological parameters in plant leaves.PRIincorporates reflectance at this band and correlates withtotal content and activity of xanthophyll cycle pigmentsand with PSII photochemical efficiency [20]. To better understand the eco-physiology of Chinese kale plants, the relationships of ChlF parameters with Chl a + b were analyzed by determining PRI.When the light intensity increased from 800 to 2000 μmol m^−2^ s^−1^, leaves with lower Chl a + b tended to show an increase in NPQ but a decrease in PRI (Figure 2). Increased NPQ values includexanthophyll cycle dependent energy quenching (photo-protection) and photoinhibitory quenching (damage of PSII) [21,22]. The consistent relationships were observed among PRI, Chl a + b, and NPQ at moderate and high PPFDs (Figure 2), suggesting that NPQ is mainly consisted of a down-regulation of PSII efficiency, whichis associated with the xanthophyll cycledependentenergy quenching, but not to photoinhibition. Therefore, it is speculated that yellow-green Chinese kale plant has high light protection abilityunder high PPFDs. Moreover, the trend of increasing NPQ values (Figure 2) is different from that of D values (Figure 1) resulted from the formulated calculations. NPQ is calculated from the maximal levels of fluorescence before (Fm) and during (Fm′) illumination in response to down-regulation of PSII. However, D is calculated from Fm′ and Fv′ in response to the fraction of light absorbed in PS II utilized in photosynthetic electron transport under illumination.

In order to characterize the photosynthetic capacity of plants, the light-response relations and consequent base points produce very important parameters. Assessing these parameters under light intensity variations provides important tools for understanding how to improve the photosynthetic productivity of plants. The analysis of ChlF and PRI combination cansearch for the photosynthetic responses of *B. oleracea* diversity that can help to explore the most suitable species composition and the structure of stands to reduce the environmental stress impacts and climate changes on these species [23]. In our study, the leaves with very lower Chl contents had higher *p* values and lower PRI values under 2000 μmol m^−2^s^−1^ PPFD conditions for 20 min, 1 h, and 2 h, suggesting that the photoinhibition did not take place, possibly resulted from the higher values of Car/Chla + b.

Light intensity canaffect the accumulation of pigments like carotenoids (Cars) and Chla + b by stimulating the enzymatic activity of kale plants subjected to light-induced stress [24]. High light intensities often generate excess heat that must be removed from photosynthetic systems to prevent pant damages. Antenna pigments, like Car, absorb light and transfer this energy to Chla + b, which initiates the sequence of photochemical events of photosynthesis [25]. Cars also channel energy away from Chla + b as a photoprotectant [26]. In our study, Cars were important for energy dissipation, and the different cultivars exhibited individual abilities and specificities of ChlF in response to PPFDs. As a result, different genotypes show different responses, and they can be used as a PPFD-plant model to investigate pigment composition under PPFD exposure. Cars function as photo-sensitizers and play important roles as scavengers of ROS.

Figure 1, Figure 2 and Figure 3 illustrate that the effects of varying light intensities on their ChlF components and PRI in regard to photosynthetic activity differed, and moderate PPFDs favored dark-green plants while yellow-green plants were adapted under high light intensities. The trend of ETR and *p* values are similar, suggesting that species can be grown under specific and optimal light intensity. The genotypic differences might be related to adaptation mechanisms induced by varying light intensities. Moreover, the impact of fertilizer treatments (FF and RF) on the physiological characteristics of photosynthesis was evaluated to quantify whether FF or RF levels affected ChlF and PRI, and results showed that fertilizer treatments did not influence the relationships both ChlF and PRI vs. Chla + b in any cultivar at all PPFD conditions. Differing responses in leaf pigments for optimizing plant growth and development in a controlled-irradiance setting depended on the cultivar of Chinese kale. Various light intensity culture systems may be used to satisfy commercial requirements for rapid, large-scale, and precise management of *B. oleracea* plant production. In addition, the average time required to measure ChlF and PRI isvery short. This means that many hundreds of individual plants can be screened per day, providing ample opportunity for the discovery of individuals that exhibit greater seedling quality. Chinese kale is an open pollinated plant that easily outcrosses with other crops, resulting in uncertain quality. Simple evaluations of photosynthesis can be made and relationships betweenheat dissipation, photosynthetic efficiency, and fluorescence can also be estimated. This knowledge could also be used in a breeding program resulting in the development of new cultivars adapted to high light intensity locations. Currently, we are using ChlF and PRI values to select for photosynthetic capacity in plants for stress tolerance.

## 4. Materials and Methods

### 4.1. Plant Materials and Cultural Practice

Seeds of Chinese kale (*Brassica oleracea* L. var. *alboglabra* Musil.) yellow-greenleaf (cv. Huang Chiehlan), greenleaf (cv. Lu Chiehlan), and dark-greenleaf (cv. HeiChiehlan) were purchased from Mingfeng (Fengyuan, Taiwan), Fangyuan (Yunlin, Taiwan), and Known-You (Kaohsiung, Taiwan) Seed Co., respectively, for our experiments. The medium usedwas a commercial potting mix of sand, peat moss, and Perlite 1:1:1 (*v*/*v*/*v*) (Known-You Co., Taipei, Taiwan). Seeds were germinated andgrown on plastic plug trays for 8–10 days until seedlings were 3 to 5 cm in height. Seedlings were then transplanted into free-draining pots (20 cm diameter, 15 cm depth, one plant per pot) and grown in a greenhouse at National Chung-HsingUniversity, Taichung, Taiwan (24°08′N, 120°40′E), during October–January in a controlled greenhouse. Plants were evenly spaced to promote similar growth rates and sizes and received regular water and fertilizers. Plants were grown for two weeks and 12 uniformly sized plants of each variety were selected and randomly separated into two fertilization groups for subsequent experiments. The full fertilization (FF) treatment comprised six plants of each variety treated with the full amount (100 mL) of a liquid fertilizer solution of 0.1% NH_4_NO_3_ and K_2_HPO_4_ twice weekly for two weeks. The restricted fertilization (RF) treatment consisted of a second batch of six plants given 0.1% NH_4_NO_3_ and K_2_HPO_4_ (50 mL) once weekly for two weeks.

### 4.2. Determination of ChlFVariables

The potted plants were moved to a dark room overnight and the middle portions of fully expanded young leaves of a plant were used for measurements. Dark-adapted plants were exposed to light stepwise from low to high levels of photosynthetic photon flux density (PPFD; i.e., 200, 400, 800, 1200, and 2000 μmol m^−2^ s^−1^) from a slide projector with a tungsten halogen lamp for 20 min and an extra 1 h and 2 h for 2000 μmol m^−2^ s^−1^ only. The ChlF parameters of dark-acclimated all night (before sunrise), light-exposed leaves, and dark-adapted for 20 min (after illumination) were measured at ambient temperature with a portable fluorometer (PAM−2000, Heinz Walz, Effeltrich, Germany). Samples were first adjusted to dark conditions to assure that all reaction centers were in an open state under minimal non-photochemical dissipation of excitation energy [27]. The values of minimal ChlF (Fo) and maximal ChlF (Fm) were respectively determined from overnight dark-adapted samples using modulated irradiation via a weak light-emitting diode beam (measuring light) and saturating pulse.For leaves under each level of illumination, the efficiency of open PSII units during illumination (Fv′/Fm′) was calculated as (Fm′−Fo′)/Fm′, and the actual PSII efficiency (ΔF/Fm’) was calculated as (Fm′−Ft)/Fm′. Fo′, Fm′ and Ft are the minimal, maximal and steady-state levels of fluorescence during each level of illumination, respectively. The former was measured after far-red illumination, middle was measured by applying a saturating flash, and the latter was determined at each PPFD level. From these data, several parameters can be computed based on modulated fluorescence kinetics [15,28]. The non-photochemical quenching (NPQ) coefficient is NPQ = (Fm − Fm′)/Fm′, *p* = ΔF/Fm′, D = 1 − (Fv′/Fm′), and E = 1 − *p* − D. *p* is the fraction of light absorbed in PSII utilized in photosynthetic electron transport. D is the fraction of light-absorbed excitation energy in PSII that is dissipated via thermal energy dissipation; *E* is the fraction of excess energy in PSII. Electron transport rate (ETR) was calculated as ΔF/Fm′ × PPFD × 0.5 × α [8]; α is the value of leaf absorbance was measured with a portable narrow-bandwidth spectra-radiometer (CI-700, Inc., Vancouver, WA, USA) in a light bench.

### 4.3. Determination of the Spectral Reflectance, Chl a + b and CarContent

Spectral reflectance was measured from the same leaves as ChlF measurement, using CI-700spectra-radiometer (CID Inc., Vancouver, WA, USA). Photochemical reflectance index (PRI) was calculated from the reflectance spectrum as (R531−R570)/(R531 + R570) for assessing xanthophyll cycle pigments [29].

Chla + b and Car contents in mature, healthy, and fully expanded middle to upper leaves were determined using methods described previously [30]. In brief, leaf discs were excised using a standard hole punch, immediately sealed in pre-labeled aluminum envelopes, and placed in liquid nitrogen. Tissues were stored at −80 °C until analysis, and then extracted in a solvent mixture of acetone, methanol, and water (80:15:5, *v*/*v*/*v*) at 4 °C overnight. The mixture was centrifugedat 13,000× *g* for 10 min, and the supernatants were then measured by a spectrophotometer (U-2000, Hitachi, Tokyo, Japan) to determine the absorbance of Chla and Chlb in acetone at 663 and 647 nm, respectively. The concentration ofChla, Chlb, and Car was calculated using the following equations:

Chla = 0.01373 × OD663 − 0.000897 × OD537 − 0.003046 × OD647, 
Chlb = 0.02405 × OD647 − 0.004359 × OD537 − 0.005507 × OD663, 
Car = [OD470 − (17.1 × (Chla + Chlb) − 9.479 × Ant)]/119.26.

### 4.4. Statistical Analysis

All experiments were arranged in a completely randomized design. All parameters were subjected to a one-way analysis of variance (ANOVA) with a significance level of *p* ≤ 0.05 using CoStatstatistical software (Cohort Berkeley, Monterey, CA, USA). Regression analyses were used to examine relationships among Chla + b and Car. In addition, model datasets were based on at least 36 leaves from each PPFD level and ChlF parameters were calculated using Chla + b data from the model validation datasets. Several models were tested, including the linear and non-linear regression models being selected forthe interpretation of the relationship between ChlF parameters and PPFD. All models were evaluated for goodness of fit by the graphical analysis of residuals and by computing correlation coefficients (r). Each experiment was performed twice independently with a randomized design for the growth environment, sampling day, and ChlF analyses.

## 5. Conclusions

There are many leaf color cultivars in Chinese kale, including green, yellow-green, and dark-green and ChlF components and PRI were used to indirectly measure the different functional levels of photosynthesis on these leaves. We showed that different cultivars of Chinese kale displayed variations in their photosynthetic apparatus associated with Chla + b and Car contents in a PPFD response, in that yellow-green cultivars displayed remarkably lower Chla + bcontents under all PPFD and fertilizer treatments. The relationships of ChlF and PRI parameters with Chl a + b contents under PPFD variations were established and can be used to improve the photosynthetic productivityand provide for eco-physiological research in Chinese kalespecies. Yield and cost are the two most important criteria in agricultural production where environmental factor optimization is concerned. The final goal of our project is to develop a new light irradiance apparatus optimized forChinese kale production in plant factories. This study provides a better understanding of the photosynthetic characteristics of Chinese kalefor promoting the rapid, large-scale, and effective management and cultivation of these plants. These results also provide deeper insight into the interception of light by photosynthetic and photo-protective pigments as a function of light intensity conditions, which is important for plant biology, as well as the knowledge-driven selection of light irradiances for non-invasive pigment estimations.
